# Eosinophilic Patterns in Patients with Seasonal Allergy Affected by Bronchial Asthma and Rhinitis/Rhinosinusitis: Efficacy of Benralizumab in Patients with the Persistent Pattern

**DOI:** 10.3390/jcm13030754

**Published:** 2024-01-28

**Authors:** Valentina D’Aiuto, Ilaria Mormile, Francescopaolo Granata, Antonio Romano, Francesca Della Casa, Caterina D’Onofrio, Valentina Marzio, Gabriele Mignogna, Luigi Califano, Amato de Paulis, Francesca Wanda Rossi

**Affiliations:** 1 Department of Translational Medical Sciences, University of Naples Federico II, 80131 Naples, Italy; valedaiuto@tiscali.it (V.D.); frapagra@hotmail.com (F.G.); francescadellacasa4@gmail.com (F.D.C.); depaulis@unina.it (A.d.P.); francescawanda.rossi@unina.it (F.W.R.); 2 Head and Neck Section, Department of Neurosciences, Reproductive and Odontostomatological Science, University of Naples Federico II, 80131 Naples, Italy; romano.antonio1972@gmail.com (A.R.); luigi.califano@unina.it (L.C.); 3 Post-Graduate Program in Clinical Immunology and Allergy, University of Naples Federico II, 80131 Naples, Italy; caterina-donofrio@libero.it (C.D.); marzio.vale@gmail.com (V.M.); gabrymig@gmail.com (G.M.); 4 Center for Basic and Clinical Immunology Research (CISI), WAO Center of Excellence, University of Naples Federico II, 80131 Naples, Italy

**Keywords:** allergic rhinitis, asthma, benralizumab, chronic rhinosinusitis, chronic rhinosinusitis with nasal polyposis, eosinophils, interleukin-5

## Abstract

Background: Eosinophilia can be influenced by multiple factors. This study aims to set a protocol for monitoring blood absolute eosinophil count (AEC) in patients with seasonal allergy affected by bronchial asthma (BA), allergic rhinitis (AR), or chronic rhinosinusitis with or without nasal polyposis (CRSw/sNP). Methods: We planned a total of four annual blood samples to measure AEC in- and out-seasonal pollen exposure (i.e., one measurement every three months for one year). Results: We identified two distinct groups of patients (non-eosinophilic and eosinophilic). Patients in the eosinophilic group presented with four different patterns (episodic, transient, floating, and persistent). Most patients with episodic, transient, and floating patterns were affected by mild allergy and the increase in eosinophils was related to allergen exposure. In contrast, patients with the persistent pattern mostly presented with more severe allergy (i.e., severe BA and relapsing CRSwNP) and the eosinophilia was unrelated to allergen exposure. The subgroup of patients with severe BA, relapsing CRSwNP, and persistent eosinophilc pattern were treated with benralizumab, which induced a noteworthy improvement in both severe BA and CRSwNP. Conclusions: Multiple AEC measurements in patients with seasonal allergy can better reflect patient’s eosinophilic status and help define the relationship of AEC enhancement with allergen exposure.

## 1. Introduction

Eosinophils are polymorphonuclear cells that differentiate in bone marrow from CD34+ pluripotent progenitor stem cells [1]. The progression of immature eosinophils towards mature cells depends on transcriptional factors and cytokines [2]. The transcriptional factor PU.1, expressed in hematopoietic cells, synergistically works with the transcriptional factors GATA-1 and CCAAT (c/EBP) to regulate the differentiation of eosinophils and the transcription of their granule proteins [2,3]. GM-CSF, IL-3, and IL-5 promote the maturation of eosinophils from myeloid precursors [3,4]. However, IL-5 is the most specific cytokine for eosinophils, influencing their differentiation, activation, migration, and survival in tissues [3,5]. In addition to cytokines, eosinophils also respond to numerous chemokines—in particular, eotaxin-1, -2, and -3; macrophage inflammatory protein (MIP)-1α; and RANTES (regulated upon activation, normal T cell expressed and secreted) [3,6]. Mature eosinophils are released from bone marrow in peripheral blood, where they have an average half-life of 8–18 h [7]. In both inflammatory and homeostatic conditions, eosinophils finally migrate to tissues such as lung and skin, where they can survive up to two weeks [3,8,9].

The normal percentage of eosinophils is 1% to 6% in bone marrow and 3% to 5% in peripheral blood, equivalent to an absolute eosinophil count (AEC) of 300 to 500 cells/μL [10]. However, AEC shows both intraindividual and interindividual variability because it is influenced by intrinsic (i.e., age, sex, BMI, ethnicity, presence of comorbidities) and extrinsic factors (i.e., exposure to allergens, seasonality, use of medical therapies, smoking of cigarettes) [11]. An increase in blood eosinophils can be classified as mild eosinophilia (500–1490 cells/μL), moderate hypereosinophilia 1500–5000 cells/µL, or severe hypereosinophilia (> 5000 cells/μL) [12,13]. Eosinophilia can be episodic, transient, floating, or persistent [13]. The causes of eosinophilia are various and can be synthesized by the acronym “APLV,” which stands for allergic disorders, parasitic infections, leukemia/lymphomas (and solid tumor), and vasculitis–immunodeficiency diseases [14]. The most common cause in industrialized nations is allergic disorders [6,15,16].

In the last few years, eosinophils have been attracting attention because of their involvement in the pathogenesis of severe forms of bronchial asthma (BA) [16] and the availability of monoclonal antibodies for the treatment of eosinophilic asthma [17]. According to the Global Initiative for Asthma (GINA) and the European Academy of Allergy & Clinical Immunology (EAACI) guidelines, eosinophilic asthma is defined by the presence of any of the following: AEC > 150 cells/µL, the presence of > 1% eosinophils in the sputum, or the presence of > 20 ppm fractional exhaled nitric oxide (FeNO) [17,18]. Of these, eosinophil count in the sputum and quantification of FeNO are not routinely used for management of asthma, whereas AEC can be easily performed and repeated in clinical practice. Currently, two anti-IL-5 therapies (e.g., mepolizumab and benralizumab) are approved in Italy as adjunctive agents for the treatment of severe eosinophilic asthma (SEA). Mepolizumab is an anti-IL5 monoclonal antibody that reduces bone marrow and airway eosinophils by approximately 50% [19] and sputum eosinophils in a dose-related way [20]. In contrast, benralizumab is an anti-IL-5Rα monoclonal antibody that ensures the near-complete depletion of eosinophils in blood and peripheral tissues via at least three mechanisms: antibody-dependent cell-mediated cytotoxicity (ADCC), antibody-dependent cellular phagocytosis (ADCP), and tumor necrosis factor (TNF)-α dependent macrophage cytotoxicity [21,22,23].

Despite the importance of AEC determination in clinical practice, a standardized protocol for monitoring AEC in allergic and non-allergic diseases is not currently available. There is only one indication that applies to persistent hypereosinophilia, which is currently defined as AEC > 1500/μL recorded on at least two occasions with a minimum time interval of two weeks [13]. Current guidelines for eosinophilic asthma are set at AEC ≥ 150 cells/μL recorded in at least one determination [17,18]. However, this threshold is debated because an AEC of ≥150 cells/μL includes individuals with AEC in the normal range (300 to 500 cells/μL, [10]). In addition, recent studies performed on large cohorts indicated that the threshold of AEC identifying eosinophilic individuals is higher than ≥ 150 cells/μL in the general population (≥ 210 cells/μL [24] and ≥ 280 cells/μL [25]) and asthma patients (≥ 384 cells/μL [25]). Finally, it is unclear whether a single AEC can be used as a reliable marker for eosinophilic asthma [11,26].

This study aims to set a protocol for monitoring the trend of blood eosinophils over a 12-month period in patients affected by BA, allergic rhinitis (AR), or chronic rhinosinusitis with or without nasal polyposis (CRSw/sNP) with seasonal allergy to parietaria and grasses.

## 2. Materials and Methods

### 2.1. Patients

A total of 78 patients with bronchial asthma (mild to moderate or severe asthma) and AR or CRSw/sNP were enrolled. Inclusion criteria were age ≥ 18 years; data available on sex, date of birth, and age of onset; and a signature on the written informed consent. In Southern Italy, most seasonal allergy at all ages (children, adults, and elderly) is mainly due to sensitization to parietaria and/or grass pollen [27,28]; therefore, we enrolled only patients sensitized to both allergens. Exclusion criteria were sensitization to perennial allergens (e.g., house dust mites, animal dander, molds), other know pulmonary diseases (e.g., chronic obstructive pulmonary disease, interstitial lung disease), use of certain medications (e.g., β-blockers, angiotensin-converting enzyme inhibitors), and uncontrolled gastroesophageal reflux disease [29,30,31,32].

At enrollment, we collected information about symptom duration, presence of eliciting triggers factors (e.g., physical effort), circadian variations, active and passive smoking, comorbidities, family history (asthma/allergic diseases), professional or private stress factors, tolerance to cyclooxygenase (COX) 1 inhibitors, response to specific therapies for asthma and nasal symptoms, long-term medication, adherence and inhalation technique, and exacerbations/hospitalizations in the previous 12 months.

Follow-up visits were scheduled every 3–4 months for the first year. After the diagnostic assessment, each patient received medical indications and therapy for the control of asthma (according to the GINA guidelines) [33] and nasal symptoms (according to the Allergic Rhinitis and its Impact on Asthma (ARIA) guidelines) [34]. Patients who experienced uncontrolled symptoms and recurrent exacerbations requiring courses of oral corticosteroids (OCSs) despite optimized standard pharmacological treatment were diagnosed as having severe bronchial asthma (SBA) according to the GINA guidelines [33], and patients with severe eosinophilic asthma (SEA) underwent therapy with anti-IL-5Rα monoclonal antibody benralizumab (see below).

All procedures performed in this study were in accordance with the ethical standards of the study center and with the 1964 Helsinki Declaration and its later amendments or comparable ethical standards and was approved by the Ethics Committee of the University of Naples Federico II (protocol code 75/21 of 7 July 2021). All the subjects enrolled gave informed consent to participate in the study. Patients undergoing treatment with benralizumab were monitored for 12 months through scheduled outpatient visits: at baseline (T0) and after 6 (T6) and 12 (T12) months.

### 2.2. Clinical Scores

The ACT (asthma control test), the VAS-ASTHMA (Asthma Visual Analog Scale), the VAS-CRS (Chronic Rhinosinusitis Visual Analog Scale), and the SNOT-22 (Sinonasal Outcome Test) questionnaire were used to measure the symptoms of bronchial asthma, rhinitis, or CRSw/sNP at the baseline and during the follow-up visits. The ACT is a five-item questionnaire that assesses asthma control over the four weeks prior to the test [35,36]. The VAS-ASTHMA and the VAS-CRS are widely used psychometric measurement tools for the assessment of asthma-related symptoms and nasal symptoms, respectively, in chronic rhinosinusitis. The VAS-ASTHMA evaluates five aspects of the pathology (dyspnea, wheezing, mucous hypersecretion, chest tightness, cough) [37]. The patient indicates the level of severity by marking a point on a straight line that corresponds to each of the five aspects (score range 0–50). The VAS-CRS has the same mode of use and investigates five nasal symptoms: sneezing, rhinorrhea, nasal obstruction, loss of smell, and headache (score range 0–50) [38,39]. The SNOT-22 is a self-administered questionnaire with 22 items to evaluate the symptoms and the impact on QoL in patients with CRSwNP [40].

### 2.3. Skin Prick Test, Total and Specific IgE

At baseline assessment, the patients carried out allergen skin prick tests (SPTs). The SPTs were performed in accordance with the EAACI guidelines with the use of a standard allergen panel (Roxall Italia SRL; Rome, Italy; LofarmaSpA, Milan, Italy). The panel included the following extracts: seven pollens (Gramineae grass pollen (Gramineae mix/Phleum Pratense/Cynodon Dactilon), mugwort, wall pellitory (Parietaria Judaica/Parietaria Officinalis), olea, cypress, birch, and hazel), dander from two animals (cat and dog), two house dust mites (Dermatophagoides pteronyssinus and Dermatophagoides farinae), tree molds (Alternaria, Aspergillus, and Cladosporium), a negative control (glycerinated saline), and a positive control (histamine). A skin test response was regarded as positive if the wheal diameter was 3 mm greater than that of the glycerinated saline control. 

Total IgE and specific IgE assay (ImmunoCAP 250; Phadia, Sweden) were also performed. IgE levels were considered positive at the level ≥ 0.35 kU/L.

### 2.4. Pulmonary Function Tests

At baseline evaluation, patients underwent spirometry to investigate the presence of ventilatory deficit. In the case of an obstructive ventilatory deficit, defined according to the criteria of the American Thoracic Society (ATS) and the European Respiratory Society (ERS), the patient underwent the standard broncho-reversibility test [41,42]. Bronchodilator reversibility was defined as an increase in FEV1 of ≥ 12% and 200 mL [43,44]. During follow-up, if necessary, a control spirometry was scheduled to evaluate the progress of the therapy or for re-evaluation of disease.

### 2.5. CT-Scan, Nasal Endoscopy, ENT (Ear–Nose–Throat) Evaluation

To investigate nasal symptoms, patients underwent imaging methods (maxillofacial computed tomography (CT) scan) and ENT (ear–nose–throat) evaluation with nasal endoscopy (NE). The maxillofacial CT scan was performed to evaluate benign (i.e., nasal polyps) or malignant neoplastic lesions and congenital or acquired anatomical drainage variants (i.e., septal deviation, presence of concha bullosa, mucocele, middle turbinate with paradoxical curvature) that alter the anatomy of the lateral nasal wall and the ostio-meatal unit and that are intended for surgical evaluation. NE is a useful technique to visualize morphological anomalies of septum, turbinate, meatuses, nasopharynx, adenoids, and Eustachian tubes orifices and identify nasal polyps and meatal secretions. Finally, it also allows for the provision of information about the upper aerodigestive tract to identify indirect signs of acid reflux. The nasal polyp score (NPS) is a clinician-reported outcome measure scored after endoscopic evaluation of the nasal cavities used to describe polyps. Each nostril is scored from 0 to 4, with 0 indicating no visible nasal polyps and 4 indicating complete obstruction of the nasal cavity by nasal polyps. Combined left and right scores give a total possible score range from 0 to 8, with higher scores indicating larger nasal polyps and greater disease severity.

### 2.6. Blood Eosinophil Count

All blood samples for full blood count and AEC were taken at certified analysis laboratories in the early morning after fasting for at least 8 h and after at least 30 days from the last dose of OCS (when applicable). We referred to the pollen calendar to identify the periods of lowest and highest concentration and diffusion of pollen of parietaria and grass. During follow-up visits, four blood samples were collected (i.e., one for each season) to evaluate the effect of allergen exposure (i.e., wall pellitory (Parietaria Judaica/Parietaria Officinalis) and Gramineae grass pollen (Gramineae mix/Phleum Pratense/Cynodon Dactilon)) on AEC. A blood sample was taken in winter (January or February), when grass and parietaria pollen are absent (allergen exposure: none); a sample was taken in spring (April or May), when the pollen concentration reaches its peak (allergen exposure: high); a sample was taken in summer (July or August), when the pollen concentration is medium (allergen exposure: medium); and a sample was taken in autumn (October or November), when the pollen concentration is low (allergen exposure: low) (Table 1). For each patient, the values of four AEC samples were recorded, and at the end of monitoring, the medium AEC (MAEC) was calculated. Eosinophilia was defined as an AEC > 500 cells/μL [13].

### 2.7. Treatments

After the diagnostic assessment, each patient received personalized indications for the control of asthma and nasal symptoms according to the GINA [33] and ARIA [34] guidelines, respectively. Patients were instructed on the correct use of the devices and on hygiene and behavioral standards to reduce modifiable risk factors. Symptoms related to gastroesophageal reflux disease were treated appropriately. Benralizumab in a dose of 30 mg, administrated subcutaneously every 4 weeks for the first three doses and every 8 weeks as a maintenance therapy, was started in patients that were diagnosed with severe eosinophilic asthma (SEA). Treatment outcomes were evaluated during the follow-up visits (T0, T6, T12) though clinical (i.e., ACT, VAS-ASTHMA, SNOT-22, VAS-CRS, NPS for CRSwNP), functional (i.e., FVC, FEV1, FEV1/FVC), and laboratory (AEC) parameters. The need for OCS administration was also recorded.

### 2.8. Data Analysis

Data were summarized via descriptive analysis. Means and SD were calculated for continuous variables, whereas absolute values and frequency (percentage) were calculated for categorical variables. The assessment of the significance of the results obtained was performed with repeated-measures one-way ANOVA with AEC (winter, spring, summer, autumn) as a within-subject factor followed by Dunnett’s test (when a comparison was made to a control) or pairwise *t*-tests (when a comparison was made between each pair of groups). All analyses were performed with IBM SPSS Statistics for Mac, version 28.0.1.0. A *p*-value of less than 0.05 was considered statistically significant for all tests. 

## 3. Results

### 3.1. Absolute Peripheral Eosinophil Count Assessment and Definition of the Eosinophilic Patterns

A total of 78 patients with bronchial asthma and AR or CRSw/sNP were enrolled in this study. Clinical features of our cohort of patients are summarized in Table 2.

**Figure 1:**
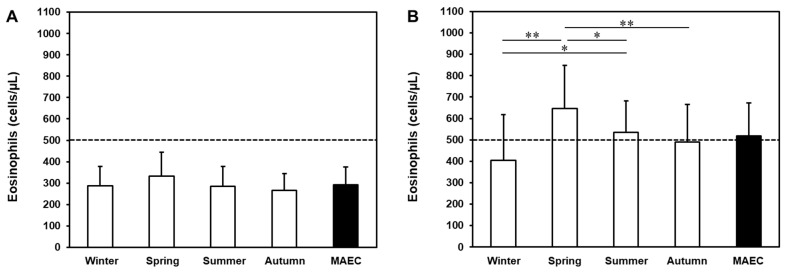
Absolute peripheral eosinophil count (AEC) in the 4 blood samples collected in the different seasons: (**A**) non-eosinophilic patients (n = 36); (**B**) eosinophilic patients (n = 42). * *p* < 0.05; ** *p* < 0.01.

To correlate AEC variations to the allergen exposure, we monitored AEC value for the four seasons (Table 1). By monitoring AEC, we identified two distinct groups of patients with seasonal allergy. In the first group (Figure 1A), defined as non-eosinophilic (n = 36, 46.2%), none of the four AEC detections was ≥ 500 cells/μL. There was no statistically significant difference in AEC among the four seasons.

In the second group (n = 42, 53.8%), defined as eosinophilic, we observed AEC ≥ 500 cells/μL in at least one of the four detections. In this group, AEC rapidly increased from the minimum value in winter to the maximum value in spring and then progressively decreased (Figure 1B). There was a statistically significant difference between AEC in spring compared to all other seasons and in summer compared to winter. There was no statistically significant difference among AEC measured in the other seasons. In addition, the MAEC of the eosinophilic group (519 ± 155 cells/μL) was significantly higher (*p* < 0.01) than the MAEC of the non-eosinophilic group (292 ± 82 cells/μL).

These results indicate that most patients with seasonal allergies showed an increase in blood eosinophils following allergen exposure, whereas the remaining patients did not show significant AEC variations throughout the monitoring period.

**Figure 2:**
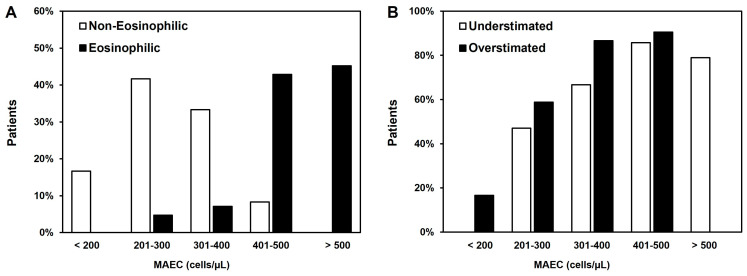
Stratification of eosinophilic (n = 42) and non-eosinophilic (n = 36) patients in categories according to medium AEC (MAEC) (**A**). Percentage of patients underestimated (step-down category of MAEC) or overestimated (step-up of category of MAEC) if a single determination of AEC (i.e., the minimum or the maximum registered value) had been considered (**B**).

We next performed a stratification of eosinophilic and non-eosinophilic patients in five categories (i.e., < 200, 201–300, 301–400, 401–500, and > 500 cells/μL) based on MAEC. Figure 2A shows that more than 90% of non-eosinophilic patients displayed an MAEC of < 400 cells/μL. In contrast, up to 90% of eosinophilic patients displayed an MAEC of > 400 cells/μL. Therefore, the threshold of 400 cells/μL appeared to be the most appropriate to discriminate eosinophilic from non-eosinophilic patients in our cohort. To challenge the usefulness of MAEC, we evaluated how many patients in each MAEC category (from both the non-eosinophilic and the eosinophilic group) could be underestimated or overestimated if a single determination of AEC (i.e., the minimum or the maximum registered AEC, respectively) had been considered. As shown in Figure 2B, across all categories, more than 50% of patients could be underestimated and/or overestimated with a single determination of AEC (mean underestimated: 69.6%; mean overestimated: 63.2%). These data support the usefulness of multiple determination of blood eosinophils and of MAEC in patient characterization.

**Figure 3:**
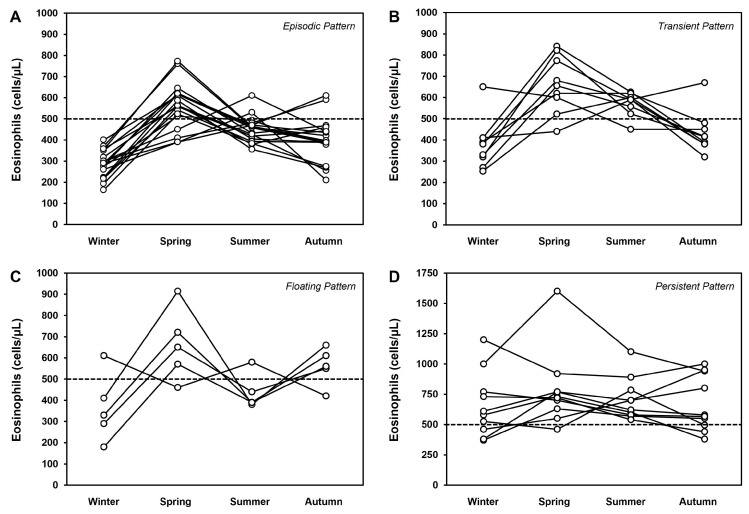
Stratification of patients in the eosinophilic group based on AEC variations and allergen exposure: (**A**) episodic pattern (n = 18); (**B**) transient pattern (n = 9); (**C**) floating pattern (n = 5); persistent pattern (**D**). See text for description of pattern characteristics.

Interestingly, when we further stratified the patients in the eosinophilic group based on AEC variations and allergen exposure, we identified four different patterns, summarized as follows:

- Episodic pattern: The number of eosinophils was > 500 cells/μL in one out of four determinations (n = 18; Figure 3A), in most cases during spring.
- Transient pattern: The number of eosinophils was > 500 cells/μL in two consecutive determinations (n = 9; Figure 3B), in most cases during spring and summer.
- Floating pattern: The number of eosinophils was > 500 cells/μL in two not-consecutive determinations (n = 5; Figure 3C); in most cases one of these was during spring.
- Persistent pattern: The number of eosinophils was > 500 cells/μL in at least three determinations (n = 10; Figure 3D), without a clear association with the seasons.

These four patterns are consistent with those previously described for eosinophilia [13].

To gain further insight into the eosinophilic pattern and allergen exposure, we next evaluated the maximum value (peak) of AEC in the various eosinophilic patterns and classified the patients in type A (i.e., peak of AEC observed in spring, allergen exposure = high) and type B (peak of AEC in another season; allergen exposure from none to medium).

As expected, in the episodic, transient, and floating patterns, most patients (n = 25; 78%) were classified as type A. Surprisingly, only a minority of patients presenting with the persistent pattern were classified as type A (n = 4; 40%), whereas most patients (n = 6; 60%) were classified as type B (Table 3). Of note, a patient with a persistent type A pattern showed an AEC of > 1000 cells/μL in the remaining seasons (Figure 3D).

We next correlated the non-eosinophilic/eosinophilic groups and patterns with patients’ clinical features (Table 3). In the non-eosinophilic group, all patients presented with mild to moderate BA and up to 90% with AR. Overall, in the eosinophilic group, most patients with the episodic, transient, or floating patterns were affected by BA, whereas AR, CRSsNP, and CRSwNP were widely distributed (20 to 44%). Interestingly, most of the patients with persistent eosinophilic pattern (n = 7; 70%) and all the patients with persistent pattern type B (n = 6; 100%) were affected by SBA and relapsing CRSwNP. Of note, the patient with persistent pattern type A and persistent AEC of > 1000 cells/μL was also affected by SBA and relapsing CRSwNP. All seven of these patients presented with similar clinical features: They showed severe eosinophilic asthma (SEA) with poor symptom control, frequent exacerbations, and relapsing CRSwNP with at least one surgery for nasal polyposis despite receiving a high dose of inhaled corticosteroids plus a second controller drug and/or OCS. Based on these findings, they were candidates for adjunctive anti-IL-5 therapy. Since our data suggest that, in these patients, persistent eosinophilia was unrelated to IL-5 pathway triggered by allergen exposure, we hypothesized that a drug able to determine complete eosinophil depletion could be more appropriate than a drug able to modulate IL-5 pathway (see the Section 4). Thus, these patients were selected for therapy with benralizumab, and their clinical features are summarized in Table 4.

### 3.2. Clinical Outcomes following Benralizumab in Patients with the Persistent Pattern

**Figure 4:**
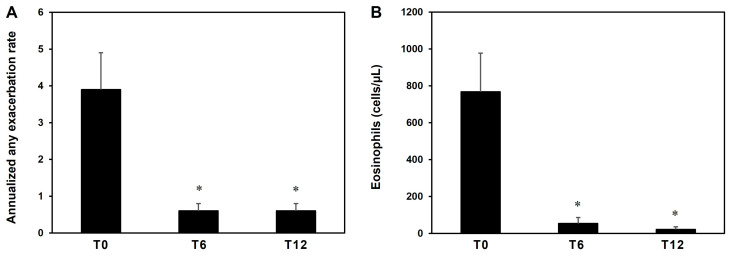
Exacerbation rate (**A**) and eosinophil count (**B**) in our cohort of patients treated with benralizumab. * *p* < 0.05.

In patients treated with benralizumab, after six months of treatment (T6), a statistically significant reduction in the number of exacerbations was observed in all patients. The data were confirmed after 12 months (T12) of follow-up (Figure 4A). AEC during therapy was significantly reduced in all patients (MAEC T0 = 768 ± 210 cells/µL vs. T12 = 21 ± 25 cells/µL; Figure 4B). No adverse reactions occurred following benralizumab administration. 

**Figure 5:**
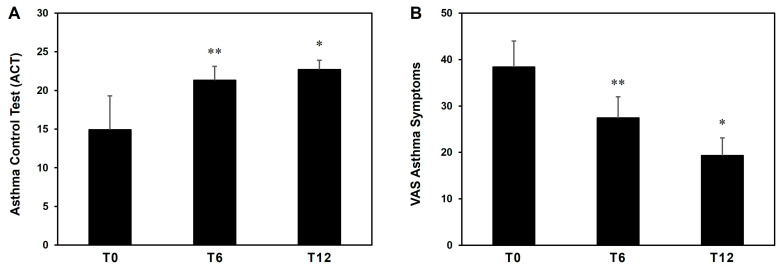
Clinical asthma outcomes (Asthma Control Test (ACT), panel (**A**) and VAS Asthma Symptoms, panel (**B**)) in our cohort of patients treated with benralizumab. * *p* < 0.01, ** *p* < 0.05.

We observed in all patients a significant improvement in both asthma control and symptoms, as assessed by ACT (mean ACT at T0 = 14.4 ± 4.7 vs. T12 = 22.7 ± 1.2; Figure 5A) and VAS-ASTHMA (mean VAS-ASTHMA at T0 = 38.4 ± 5.6 vs. T12 = 19.3 ± 3.8; Figure 5B), respectively. Both improvements were already significant at T6 (ACT = 43.0%; VAS-ASTHMA = 28.6%), with a further enhancement at T12 (ACT = 52.3%; VAS-ASTHMA = 49.7%).

**Figure 6:**
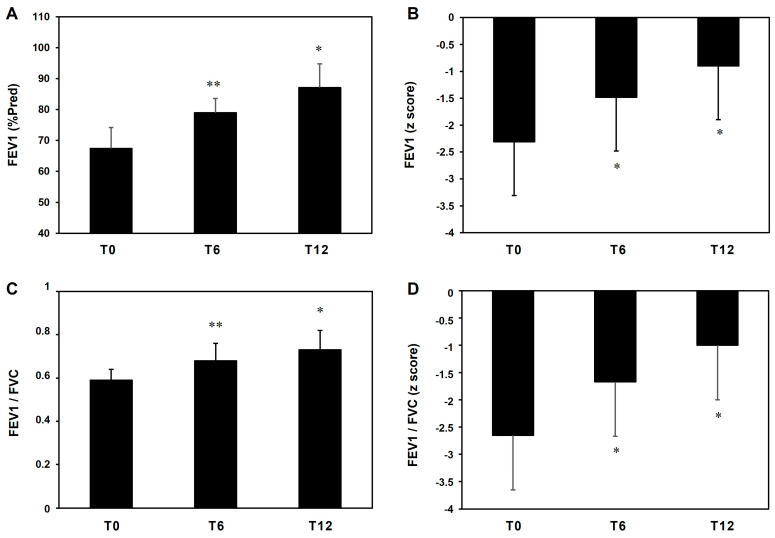
Pulmonary function tests (FEV1, panel (**A**) and (**B**) and FEV1/FVC, panel (**C**) and (**D**)) in our cohort of patients treated with benralizumab. * *p* < 0.01, ** *p* < 0.05.

This clinical improvement was confirmed by the pulmonary function parameters FEV1 (% Pred and z-score) and FEV1/FVC (ratio and z-score), which progressively improved in T6 (FEV1-%Pred = 17.2%; FEV1 z-score = 35.9%; FEV1/FVC = 15.3%; FEV1/FVC z-score = 37.0%) and in T12 (FEV1-%Pred = 29.2%; FEV1 z-score = 61.0%; FEV1/FVC = 23.7%; FEV1/FVC z-score = 62.3%) (Figure 6). 

**Figure 7:**
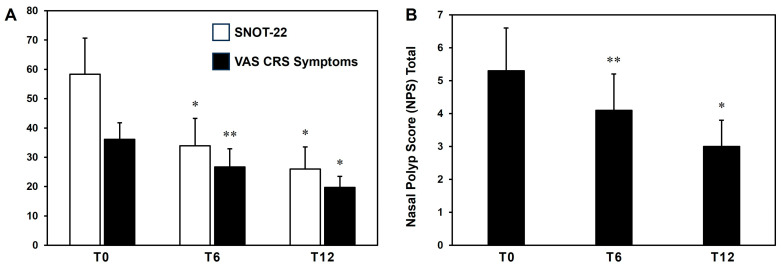
Rhino-nasal outcomes (Sinonasal Outcome Test (SNOT)-22 and Chronic Rhinosinusitis Visual Analog Scale (VAS CRS) Symptoms, panel (**A**); Nasal Polyp Score (NPS) Total, panel (**B**)) in our cohort of patients treated with benralizumab. * *p* < 0.01, ** *p* < 0.05.

Benralizumab was also effective at reducing nasal symptoms, as shown by the statistically significant improvement in SNOT-22 and VAS-CRS values at T6 (SNOT-22 = 41.9%; VAS-CRS = 26.0%) and T12 (SNOT-22 = 55.4%; VAS-CRS = 45.4%) (Figure 7A). The clinical data were confirmed by the reduction in the size of the polyps in 100% of patients at T6 (NPS = −22.6%) and T12 (NPS = −43.4%) (Figure 7B).

## 4. Discussion

In patients with seasonal allergy, the oscillation of blood eosinophils in season and out of season is well known [45,46]. In some geographical regions, including Southern Italy, seasonal allergens can present very long-lasting periods of pollination and be the major allergens responsible for BA and AR [27,47,48]. Indeed, we previously reported that Parietaria and/or Gramineae grass were the dominant allergens in 75.5% of patients from Southern Italy affected by AR and/or BA, as assessed with a multi-parameter score [27]. In our view, a single determination of AEC in these patients provides only a partial characterization of the eosinophilic status [26,49,50]. For these reasons, herein we present an eosinophil-monitoring model to be applied in patients with seasonal allergy, which represents an easy-to-use tool for clinicians and a low-cost protocol for patients. Our model, based on four determinations of AEC and calculation of MAEC, allowed the patient’s eosinophilic status to be defined better, the relationship with allergen exposure to be verified, and the impact of intraindividual variability to be reduced.

To date, eosinophilia is defined as an AEC of > 500 cells/μL in a single determination [13]. Using this threshold in our model, we initially defined two groups of patients: non-eosinophilic vs. eosinophilic. However, a MAEC of 400 cells/μL appeared to be more appropriate to discriminate between these two groups (Figure 2A). In addition, most of our patients would have been underestimated and/or overestimated with a single determination of AEC performed during a nadir or a peak in these cells, respectively (Figure 2B). Indeed, in recent studies performed in real-life settings on large cohorts of individuals, seasonal allergy was only one of several intrinsic and extrinsic factors causing intraindividual and interindividual variability in AEC in the general population as well as in the asthma population [11,24,25]. Therefore, it is conceivable that the MAEC assessment through four AEC determinations could be a more appropriate tool for managing patients with various eosinophilic conditions [11]. The simplicity of use and the versatility of our eosinophil-monitoring model make it suitable for use in all patients with allergic asthma by adapting the blood-sampling schedule based on the allergen exposure. Furthermore, four-month eosinophil monitoring can be included in the six-month evaluation period suggested by the GINA guidelines [33,51] for the diagnosis of SBA. Finally, our model could be also used for other diseases related to eosinophilia—for example, in hypereosinophilic syndrome [52,53,54] and eosinophilic esophagitis [55]—to evaluate the trend of eosinophils in response to several factors and therapy.

Thanks to the multiple determinations of AEC, in our eosinophilic group, we identified four different eosinophilic patterns that are consistent with those previously described for eosinophilia [13]. The episodic, transient, and floating patterns showed a greater correlation with the allergen exposure (Type A 77.8–80% vs. Type B 20–22.2%) whereas the persistent pattern appeared to be more frequently unrelated to the allergen exposure (Type A 40% vs Type B 60%) (Table 3). This finding suggests that the episodic, transient, and fluctuating patterns are related to IL-5 pathway triggered by allergen exposure, whereas in the persistent pattern, eosinophilia could be at least partially independent of the IL-5 pathway. This hypothesis is in line with a recent study showing the efficacy of benralizumab in late non-responders to mepolizumab [56]. In this study, the authors hypothesized that in patients non-responding to mepolizumab, eosinophilia can be mediated by other mechanisms such as activation of other cytokine pathways and/or mutations of IL-5 receptor. It is known that eosinophils express on their surface receptors for several cytokines, including epithelial cytokines, IL-3, and GS-CMF, which also play a pivotal role in type 2 (T2) inflammation and can stimulate eosinophils [16,56,57]. Several studies carried out in humans and animal models of eosinophil allergic diseases revealed that although IL-5 has a major role in eosinophil physiology [4,16,58], IL-4, IL-9, IL-13, IL-18, IL-25, and IL-31 cytokines may also play a role [57,59,60].

Current concepts support the hypothesis that SBA is the result of changes in the complex biological networks with distinct but interrelating immune–inflammatory responses that are continuously modified over time through the activation of different cytokine pathways [17]. Our patients with SBA and relapsing CRSwNP who were candidates for adjunctive anti-IL-5 therapy exhibited persistent eosinophilia. These patients also showed older age and a longer disease duration compared to the patients with mild to moderate asthma. Thus, we figured out that the persistent eosinophilia of these patients was at least partially independent of the IL-5 pathway because of the dynamic changes in the cytokine pathways possibly related to the dynamic changes in allergen exposure over time, thereby becoming the driving factor causing the severity of the asthma. Thus, we prospectively decided to treat this group of patients with benralizumab, which is a drug that can realize complete eosinophil depletion [21,61], rather than mepolizumab, which exclusively modulates the only IL-5 pathway [62].

The efficacy of benralizumab in severe eosinophilic asthma has been consistently seen in patients with an AEC of ≥ 300 cells/µL [63,64]. In addition, in a recent retrospective study performed in real-life settings on 429 patients, it has been shown that benralizumab is also able to reduce asthma exacerbations by approximately 50% in patients with an AEC of < 300 cells/µL [65]. In our patients, the MAEC was > 700 cells/µL (Table 4), and treatment with benralizumab for 12 months induced a reduction of up to 90% of the exacerbation rate. We observed a significant improvement in both asthma and CRSwNP in 100% of patients. In particular, benralizumab induced an improvement of approximately 50% of the clinical scores of asthma and nasal polyposis (ACT, VAS-ASTHMA, SNOT-22, and VAS CRS), an improvement in pulmonary function parameters from 24 to 62% (as assessed by FEV1 and FEV1/FVC), and a reduction of up to 45% in the endoscopic score of nasal polyposis (NPS). These data are in line with several retrospective studies performed in real-life settings in which benralizumab in patients with an AEC of > 600 cells/µL reduced the asthma exacerbation rate by approximately 90% and induced improvement in both asthma and CRSwNP parameters [66,67,68,69,70]. What we added to these observations with our study is the perspectival approach with which patients’ characteristics suggested the right biological drug. In fact, though our data need to be confirmed in larger cohorts and do not exclude that other biological drugs may be equally effective, they indicate that naïve patients with SEA due to exposure to seasonal aeroallergens, relapsing CRSwNP, persistent eosinophilia, and a MAEC of > 700 cells/μL can greatly benefit from treatment with benralizumab.

There is no consensus regarding the number of determinations necessary before starting biological drugs [11]. Clinical trials conducted with mepolizumab suggested that a single measurement was sufficient to guide the therapeutic choice because most of the patients enrolled remained in the same group [71,72]. In contrast, several studies conducted in real-life settings [26,49,50] showed that, due to the within-subject biological variation in eosinophil count, multiple measurements of AEC over time are necessary to better define the patient’s eosinophilic status and choose the most appropriate therapy. Our data are in accordance with this last approach. In fact, we carried out a prospective study in a real-life setting in which the eosinophil-monitoring model we applied revealed itself to be a highly effective therapeutic option.

We are aware that it will be necessary to apply the proposed monitoring model in a larger series of patients to confirm whether multiple AEC measurements can be a tool that is able to define a patient’s eosinophilic status and correlate the eosinophilic pattern with the severity of allergic diseases. A method of clustering and multivariate analysis of clinical, laboratory, and instrumental parameters in a real-life setting could allow for a better definition of the allergic phenotype and identification of early or late onsets associated with a specific eosinophilic pattern. The ability to identify biomarkers useful for defining personalized treatments is gaining importance, especially now that new pharmacological options are available. In fact, the appropriate use of biological drugs is essential both to obtain the best benefit for the patient and to optimize healthcare resources.

## 5. Conclusions

Multiple AEC measurements and calculations of MAEC in patients with seasonal allergy can better reflect their eosinophilic status and help define the relationship between AEC enhancement and allergen exposure. In our monitoring of AEC, we observed that in patients with mild allergy, the increase in eosinophils was related to pollen exposure, whereas patients with more severe allergy showed persistent eosinophilia unrelated to pollen exposure. In these last patients, treatment with benralizumab induced a significant improvement in both SBA and CRSwNP. Definition of a standardized protocol for the monitoring of AEC could be useful for identifying patients that may benefit from the novel T2 endotype-targeted therapies.

## Figures and Tables

**Table 1:** Proposed scheme for eosinophil monitoring.

	Winter	Spring	Summer	Autumn
Allergen exposure	None	High	Medium	Low
Blood drawing	January–February	April–May	July–August	October–November

**Table 2:** Characteristics of patients in our cohort with bronchial asthma (n = 78).

Patients’ Features	Mean ± SD
Female gender (n, %)	38 (48.7%)
Caucasian ethnicity (n, %)	78 (100%)
Age, years (mean ± SD; range)	41.7 ± 12.8; 18–73
BMI (Kg/m^2^)	26.3 ± 5.2
Total IgE (IU/mL)	287.4 ± 154.7
Parietaria skin prick test (mm)	15.3 ± 4.1
Parietaria-specific IgE (kU/L)	13.9 ± 8.1
Gramineae grass skin prick test (mm)	15.6 ± 3.6
Gramineae grass specific IgE (kU/L)	7.3 ± 2.9

**Table 3:** Stratification of non-eosinophilic and eosinophilic patients based on clinical and laboratory parameters (n = 78). MAEC, medium AEC. Type A: peak of AEC observed in spring; Type B: peak of AEC observed in the other seasons. BA, bronchial asthma (mild to moderate); SBA, severe bronchial asthma; AR, allergic rhinitis; CRSsNP, chronic rhinosinusitis without nasal polyposis; CRSwNP, chronic rhinosinusitis with nasal polyposis.

	**Group**		**Pattern of the Eosinophilic Group**			
	**Non-Eosinophilic**	**Eosinophilic**	**Episodic**	**Transient**	**Floating**	**Persistent**
**Number of** **Patients**	36	42	18	9	5	10
**%**	46.2%	53.8%	42.9%	21.4%	11.9%	23.8%
**MAEC ± SD**	292 ± 82	519 ± 155	425 ± 49	512 ± 38	506 ± 60	707 ± 210
**Type A (n.)**	N.A.	29	14	7	4	4
**Type A (%)**	N.A.	69.0%	77.8%	77.8%	80.0%	40.0%
**Type B (n.)**	N.A.	13	4	2	1	6
**Type B (%)**	N.A.	31.0%	22.2%	22.2%	20.0%	60.0%
	**Group**		**Pattern of the Eosinophilic Group**			
	**Non-Eosinophilic**	**Eosinophilic**	**Episodic**	**Transient**	**Floating**	**Persistent**
**Number of** **Patients**	36	42	18	9	5	10
**BA (n.)**	36	31	16	8	4	3
**BA (%)**	100.0%	73.8%	88.9%	88.9%	80.0%	30.0%
**SBA (n.)**	0	11	2	1	1	7
**SBA (%)**	0%	26.2%	11.1%	11.1%	20.0%	70.0%
	**Group**		**Pattern of the Eosinophilic Group**			
	**Non-Eosinophilic**	**Eosinophilic**	**Episodic**	**Transient**	**Floating**	**Persistent**
**Number of** **Patients**	36	42	18	9	5	10
**AR (n.)**	32	13	8	3	2	0
**AR (%)**	88.8%	30.9%	44.4%	33.3%	40.0%	0%
**CRSsNP (n.)**	2	11	6	3	1	1
**CRSsNP (%)**	5.6%	26.2%	33.3%	33.3%	20.0%	10.0%
**CRSwNP (n.)**	2	18	4	3	2	9
**CRSwNP (%)**	5.6%	42.9%	22.2%	33.3%	40.0%	90.0%

**Table 4:** Characteristics of patients with SBA + CRSwNP before treatment with benralizumab (T0) (n = 7).

Patients’ Features	Mean ± SD
Female gender (n, %)	3 (48.8%)
Caucasian ethnicity (n, %)	7 (100%)
Age, years (mean ± SD)	50.8 ± 14.2
BMI (Kg/m^2^)	28.8 ± 4
Disease duration (asthma; years)	14.4 ± 6.3
Disease duration (CRSwNP; years)	7.0 ± 4.1
Annualized any exacerbation rate	3.9 ± 1.0
Polyp Surgeries (mean ± SD)	1.4 ± 0.5
ACT (mean ± SD)	14.4 ± 4.7
VAS-Asthma (mean ± SD)	38.7 ± 5.7
VAS-CRS (mean ± SD)	36.1 ± 5.7
SNOT-22 (mean ± SD)	58.3 ± 12.4
NPS (mean ± SD)	6.4 ± 0.9
MAEC ± SD (cells/μL)	768 ± 210
Total IgE (IU/mL)	232.0 ± 111.8
Parietaria skin prick test (mm)	16.3 ± 3.2
Parietaria specific IgE (kU/L)	15.6 ± 23.9
Gramineae grass skin prick test (mm)	14.1 ± 2.2
Gramineae grass specific IgE (kU/L)	4.8 ± 2.3
FVC (% Pred; z-score)	90.8 ± 7.0; −0.63 ± 0.44
FEV1 (% Pred; z-score)	67.4 ± 6.8; −2.31 ± 0.53
FEV1/FVC ratio; z-score)	0.59 ± 0.06; −2.65 ± 0.65

## Data Availability

The data presented in this study are available on request from the corresponding author.
